# The Presence of Bilateral Dilated Pupils is Not a Death Sentence in a Stroke Patient: A Case Report

**DOI:** 10.7759/cureus.69133

**Published:** 2024-09-10

**Authors:** Mihail Petrov, Teodora Sakelarova, Nikolay Velinov, Maria Dimitrova, Nikolay Gabrovsky

**Affiliations:** 1 Neurological Surgery, University Multiprofile Hospital for Active Treatment and Emergency Medicine "N. I. Pirogov", Sofia, BGR; 2 Neurology, University Multiprofile Hospital for Active Treatment and Emergency Medicine "N. I. Pirogov", Sofia, BGR

**Keywords:** basilar artery occlusion, coma recovery, dilated pupils, endovascular treatment, ischemic stroke

## Abstract

Bilateral dilated pupils are an ominous clinical sign of brainstem dysfunction, which uniformly leads to a bad prognosis for the patient. In some rare instances in adult patients, it could be reversible. We present a clinical case of an elderly stroke patient with bilateral dilated pupils with a surprisingly favorable clinical outcome.

An 80-year-old female patient presented in the emergency department in a coma, areflexia, and bilaterally dilated non-reactive pupils. One and a half hours ago the patient suddenly lost consciousness and became unresponsive. A computed tomography (CT) scan showed a hyperdense basilar tip, and CT angiography confirmed the presence of a defect in the filling of the basilar tip and the bilateral P1 segments of the posterior cerebral arteries (PCA). The patient was ineligible for intravenous thrombolysis. Endovascular treatment was performed with partial recanalization of the basilar artery thrombolysis in cerebral ischemia (TICI) 2a.

The diameter and light reactivity of patients’ pupils are important parts of the neurological exam. A dilated pupil is an ominous sign associated with a severe prognosis and even worse if both pupils are dilated. Bilateral fixed dilated pupils could be present in basilar artery occlusion (BAO), i.e., basilar tip occlusion. This is explained by ischemia in the mesencephalon, where the nucleus of the oculomotor nerve lies. This ischemic stroke has the highest mortality rate, greater than 85%. The only proven treatment for BAO patients is recanalization with intravenous r-tPA (recombinant tissue plasminogen activator), intra-arterial r-tPA, or endovascular treatment. With adequate treatment, a good outcome can be obtained in up to 35%, and the mortality can be dropped to 40%.

Patients with posterior circulation stroke, especially BAO, are still one of the hardest to diagnose on time. They require timely and coordinated efforts by an interdisciplinary team of neurologists, neuroradiologists and neurosurgeons. Timely recanalization within 12 hours and potentially up to 24 hours is the goal. This could lead to a favorable outcome. Loss of consciousness and bilateral fixed dilated pupils could be present in patients with BAO and shouldn’t be accepted as a sign of a definite bad outcome. This definitely should not discourage treating physicians. All efforts should be focused on finding the right diagnosis in a timely manner. The differential diagnosis is crucial and may be the difference between life and death, especially in the context of BAO.

## Introduction

Bilateral dilated pupils are an ominous clinical sign of brainstem dysfunction, which uniformly leads to a bad prognosis for the patient [1]. Patients with bilaterally dilated pupils are considered to have a very grave prognosis. More frequently in the pediatric population, it could be associated with a favorable prognosis. In some rare instances in adult patients, it could also be reversible. Basilar artery occlusion (BAO) leads to devastating stroke with very high mortality and morbidity. As the ischemic stroke with the highest mortality rate, greater than 85%, the only proven treatment for BAO patients is recanalization with intravenous r-tPA (recombinant tissue plasminogen activator), intra-arterial r-tPA, or endovascular treatment. With adequate treatment, a good outcome can be obtained by up to 35%, and the mortality can be dropped to 40%. We present a clinical case of an elderly stroke patient with bilateral dilated pupils with a surprisingly favorable clinical outcome.

## Case presentation

**Figure 1:**
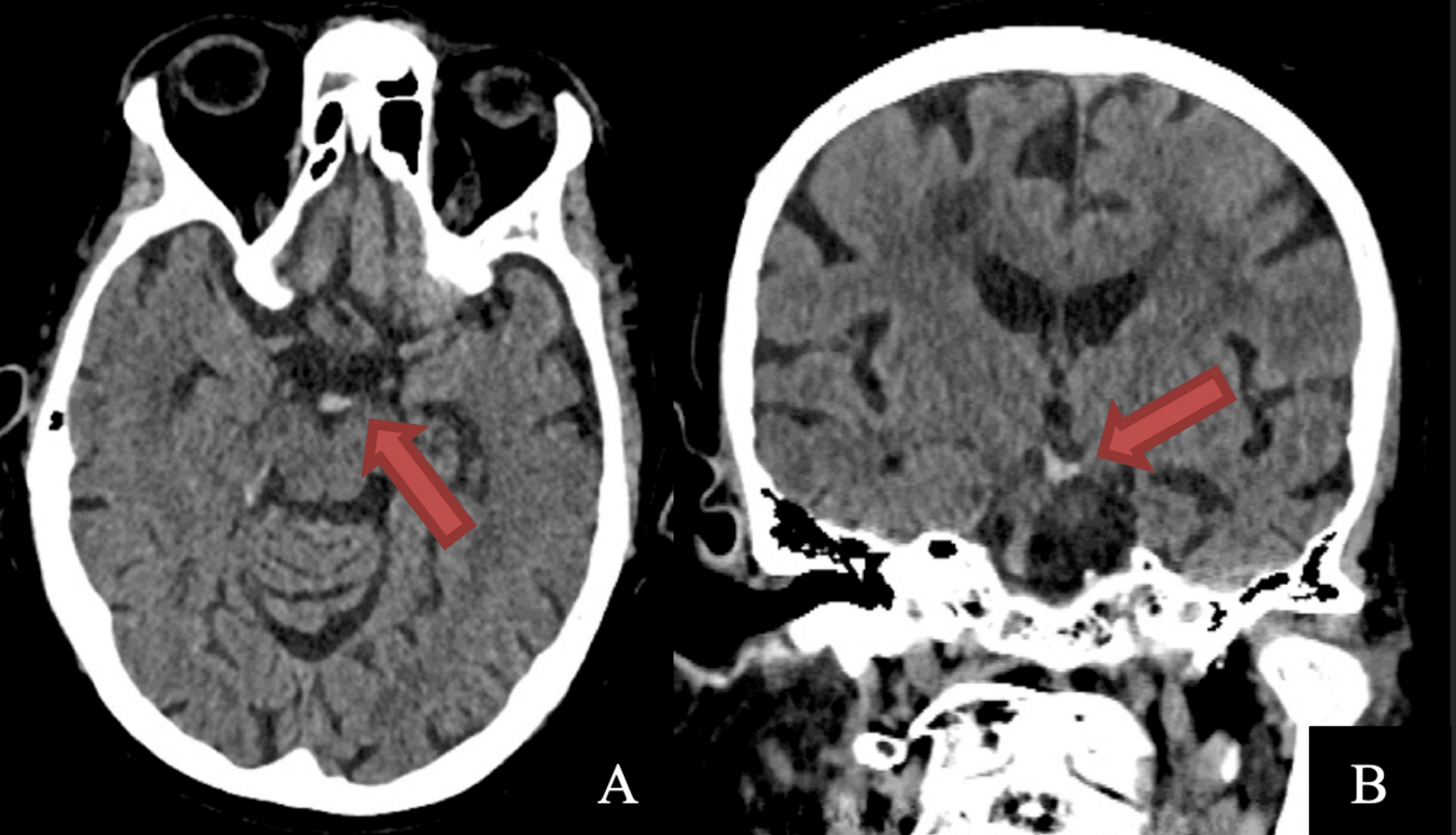
Preoperative brain CT scan of the patient (A) Axial image (B) Coronal image. A hyperdense basilar tip is evident in both images.

**Figure 2:**
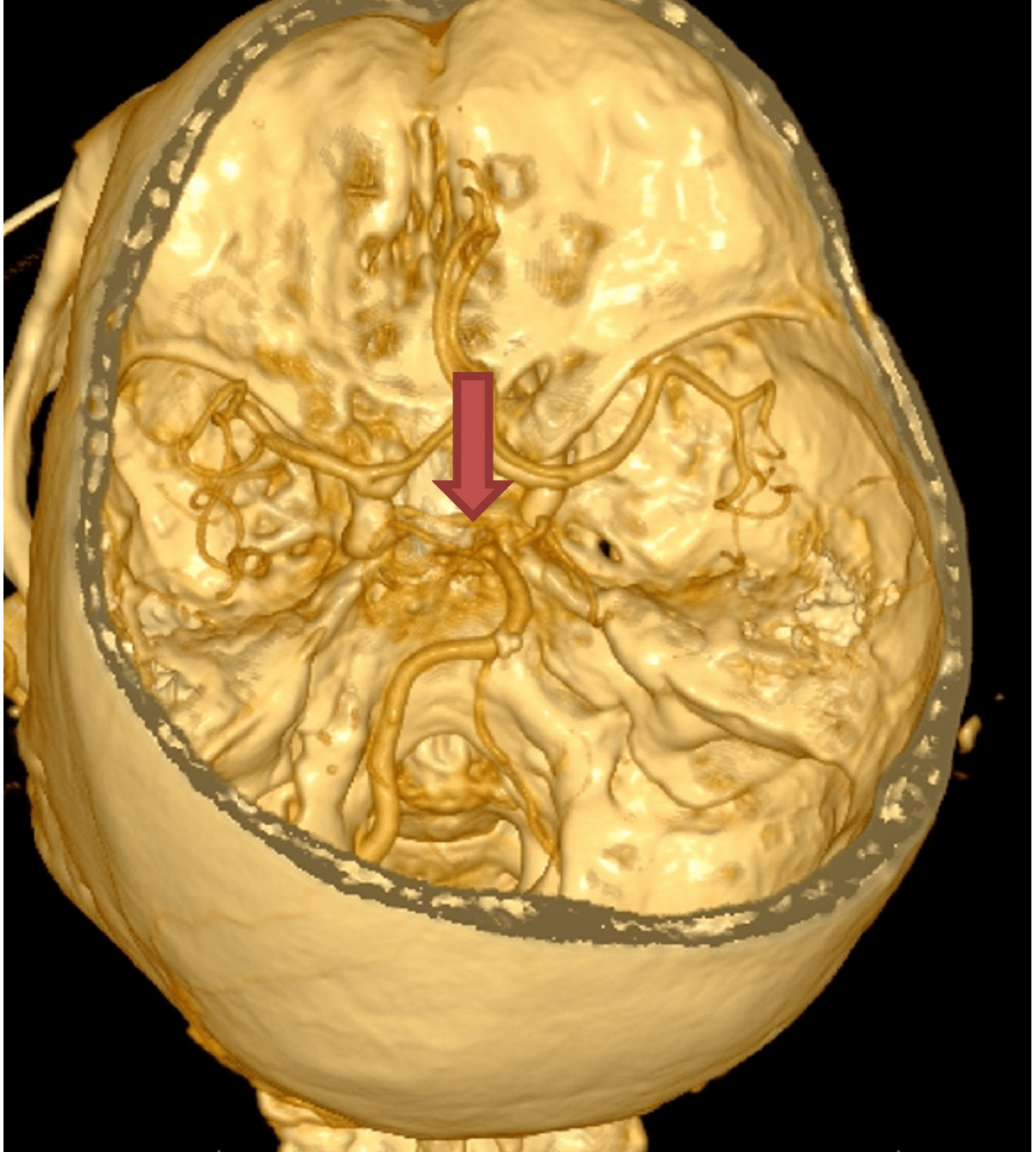
3D reconstruction of the CT-angiography A defect in the filling of the basilar tip and the P1 segments of both PCAs (red arrow).

An 80-year-old female patient presented in the emergency department in a coma, areflexia, and bilaterally dilated non-reactive pupils. The patient suddenly lost consciousness and became unresponsive one and a half hours before the presentation. A computed tomography (CT) scan showed a hyperdense basilar tip (Figure 1), and CT angiography (Figure 2) confirmed the presence of a defect in the filling of the basilar tip and the bilateral P1 segments of the posterior cerebral arteries (PCA).

**Figure 3:**
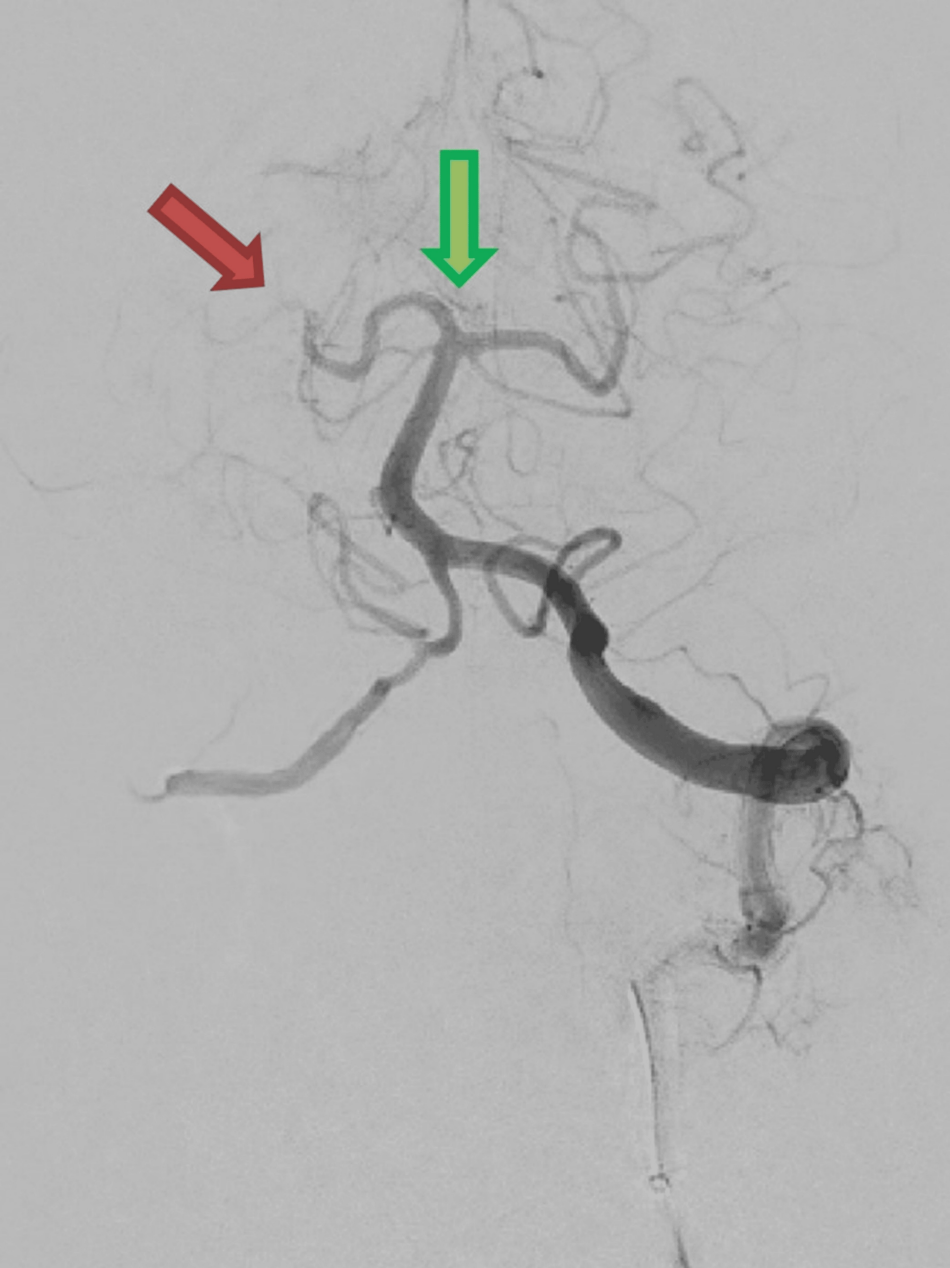
Angiography from the left dominant vertebral artery Basilar tip recanalization (green arrow) with persistent posterior cerebral artery (PCA) occlusion (red arrow) on the right.

Due to the severe status of the patient, NIHSS (National Institute of Health Stroke Scale) 30, the patient was ineligible for intravenous thrombolysis. An elaborate discussion was conducted with the relatives of the patient about the possible outcomes with and without treatment. The short interval from onset of symptoms to arrival (only 1.5 hours) was the main argument in favor of performing endovascular treatment. After obtaining informed consent, the patient was brought to the angiography suite. Arterial blood pressure was maintained as high as 200/100 mmHg. Angiographic runs confirmed recanalization of the basilar tip (Figure 3) but persistent occlusion of the right PCA.

**Figure 4:**
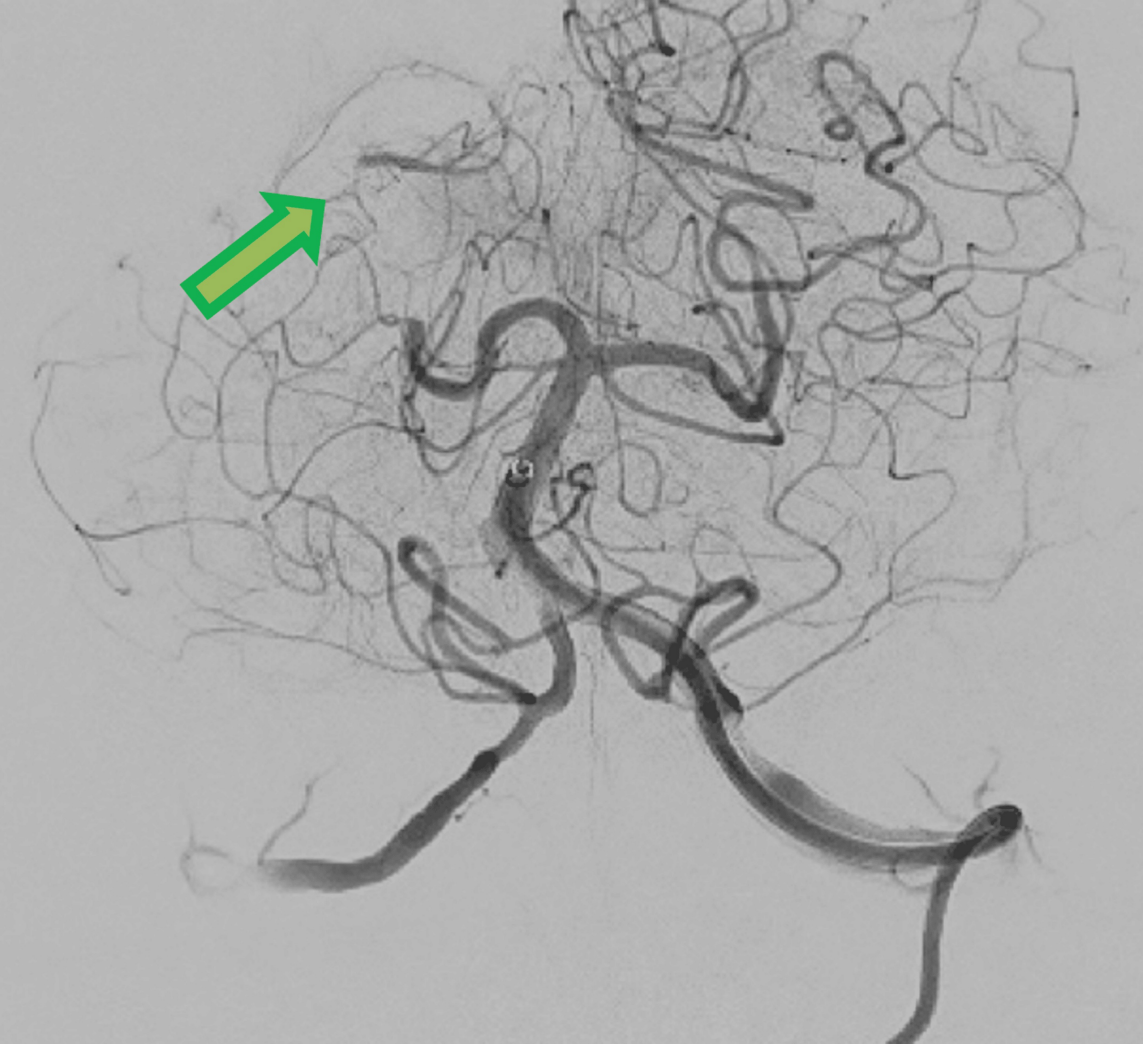
Angiography after aspiration with a 4max aspiration catheter and selective intraarterial thrombolysis in the right PCA Partial recanalization of the vessel is shown with a green arrow. Due to the severe status of the patient, more aggressive endovascular techniques were considered unreasonable. PCA: Posterior cerebral artery; 4max aspiration catheter (Penumbra Inc, Alameda, CA)

Selective direct aspiration was performed with a 4max aspiration catheter (Penumbra Inc, Alameda, CA) and selective intraarterial thrombolysis. Partial recanalization was achieved - Thrombolysis in Cerebral Ischemia (TICI 2a) (Figure 4).

**Figure 5:**
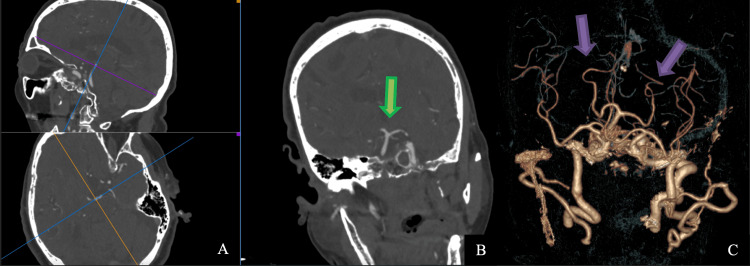
Postoperative CT-angiography images A, B: Multiplanar reconstruction (MPR) images with complete recanalization of the tip of the basilar artery, marked with a green arrow in B. C: The 3D reconstruction of the CT angiography shows perfect filling with a contrast of both posterior cerebral arteries (PCAs), marked with purple arrows.

Due to the severe status of the patient, more aggressive endovascular techniques were deemed unreasonable. Control CT and CT-angiography revealed no ischemic changes and complete patency of the basilar artery (Figure 5).

Two days later, the patient was awake and extubated. There was no motor deficit in the limbs, with persistent bilateral ptosis and external and internal ophthalmoparesis. The patient was discharged on 10th postoperative day ambulating with assistance and referred to an ophthalmologist to correct the ptosis at least unilaterally. The latter would allow the patient to see and walk on her own.

## Discussion

The diameter and light reactivity of patients’ pupils is an important part of the neurological exam. A dilated pupil is an ominous sign associated with a severe prognosis and even worse if both pupils are dilated. It should be borne in mind that the application of some medications could lead to bilateral mydriasis [2]. Patients with bilaterally dilated pupils have a more grave prognosis than patients with a single dilated pupil [1]. However, the studies of patients with bilateral dilated pupils are mainly in the field of traumatic brain injury (TBI), intracerebral hemorrhage, or after spontaneous subarachnoid hemorrhage (SAH) [3]. In the study by Clusmann et al. [3], of 99 patients included, only 10% reached favorable outcomes, and the mortality rate was 75%. None of these patients had an ischemic stroke. No such studies have been conducted so far. 

Pupil dilation is due to compression of the lateral aspect of the oculomotor nerve. It is usually due to intracranial space occupying a lesion that leads to raised intracranial pressure and cerebral herniation. In the context of ischemic stroke, this usually occurs late in the evolution of brain ischemia when malignant cerebral edema is present, for instance, 72 hours after middle cerebral artery occlusion. However, bilateral fixed dilated pupils could be present in basilar artery occlusion, i.e., basilar tip occlusion. This could be due to ischemia in the mesencephalon where the nucleus of the oculomotor nerve lies (which is accompanied by symptoms due to damage of other tracts, not present in the described case) or to an infranuclear lesion of the oculomotor nerves. With magnetic resonance imaging /MRI/ the mesencephalic lesion could be visualized. 

In carefully selected patients, treatment should not be denied as favorable outcome is possible. Stroke patients with basilar artery occlusion are perfect example for patients that could benefit from endovascular treatment and reach meaningful functional recovery. 

Basilar artery occlusion is a devastating stroke with very high mortality and morbidity. It is found in up to 27% of ischemic strokes occurring in the posterior circulation and in about 1% of all strokes [4]. Most commonly, basilar artery occlusion patients present with acute neurologic symptoms, including motor deficits, hemi- or quadriparesis, cranial nerve palsies, speech abnormalities, and decreased level of consciousness. In more than 40% of these patients, pupillary abnormalities, oculomotor signs, and pseudobulbar manifestations can be observed [5]. As the ischemic stroke with the highest mortality rate, greater than 85%, the only proven treatment for BAO patients is recanalization with intravenous r-tPA, intra-arterial r-tPA, or endovascular treatment. With adequate treatment, a good outcome can be obtained in up to 35%, and the mortality can be dropped to 40% [4]. There is still no definite consensus on the treatment window as there are still few large-scale randomized trials focusing on BAO. It is though commonly acceptable to recanalize in at least 12 hours and potentially up to 24 hours [6]. Therefore, in patients presenting with loss of consciousness and oculomotor symptoms, and especially fixed pupillary dilatation, the differential diagnosis is crucial and time-sensitive.

## Conclusions

Patients with posterior circulation stroke, especially BAO, are still among the hardest to diagnose on time. They require timely and coordinated efforts by an interdisciplinary team of neurologists, neuroradiologists, and neurosurgeons. Timely recanalization within 12 hours and potentially up to 24 hours is the goal. This could lead to a favorable outcome.

Loss of consciousness and bilateral fixed dilated pupils shouldn’t be accepted as a sign of a definite bad outcome. This should definitely not discourage treating physicians. All efforts should be focused on finding the right diagnosis in a timely manner. The differential diagnosis is crucial and may be the difference between life and death especially in the context of BAO.
